# Adult attachment and dehumanization among Chinese college students: trait attachment and state attachment perspectives

**DOI:** 10.3389/fpsyg.2024.1453855

**Published:** 2024-09-19

**Authors:** Minna Guo, Beibei Xu, Haiyao Wang, Thi Quynh Mai Le, Zhihao Yan, Qingsong Sang

**Affiliations:** School of Education Sciences, Anhui Normal University, Wuhu, China

**Keywords:** dehumanization, adult attachment, trait attachment, state attachment, latent profile analysis

## Abstract

**Introduction:**

Current social issues such as bullying, online violence, and local conflicts are all prominent topics in the field of psychology and linked to dehumanization. However, research on dehumanization from a non-Western background has been rare. As a key factor influencing individual behavior, attachment has not been sufficiently integrated into studies on dehumanization. Therefore, this study provides empirical evidence to explore the relationship between adult attachment (both trait and state) and dehumanization. The sample consisted of college students from China.

**Methods:**

In Study 1 (*N* = 705) latent profile analysis was employed to exploring the potential categories of trait adult attachment in China, and to investigate how these categories impact dehumanization. Study 2 (*N* = 281) focused on activating secure attachment and examined the effects of three state attachment types, security, avoidance and anxiety, on dehumanization.

**Results:**

Study 1 identified four types of adult attachment: preoccupied, secure, fearful, and dismissing, and revealed that these different attachment types have varying impacts on dehumanization. Study 2 showed that both security and avoidance predict dehumanization.

**Discussion:**

This research established a link between adult attachment and dehumanization, offering new insights into the psychological mechanisms underlying dehumanization and suggesting novel strategies for its prevention and intervention.

## 1 Introduction

Psychologists have long been concerned with social issues such as violence, racial discrimination, racial stereotypes, and the imitation of violent game behaviors. A possible explanation for these phenomena involves the denial of human nature, a process called dehumanization (Haslam, 2021). Exploring dehumanization is valuable for understanding and analyzing negative social behaviors and their underlying psychological mechanisms. Over the past 2 decades, dehumanization has gained increased attention (Haslam, 2022). Researchers have explored the potential outcomes of denying or ignoring human nature, such as reduced prosocial behaviors and increased antisocial behaviors. However, there is still a lack of research addressing the variables that trigger or hinder dehumanization (Ariño-Mateo et al., 2024).

It is worth noting that dehumanization varies across different cultures (Arriagada-Venegas et al., 2022), but research on dehumanization in Chinese culture and cross-cultural studies remains limited. Existing studies in China primarily focus on topics such as the stability of power and the impact of prosocial video games on dehumanization perceptions (Chen et al., 2014; Sun and Liu, 2021).

This gap in research May arise from differing views on human nature in Western and Eastern cultures. In Western cultural contexts, there is a dominant belief in the inherent malignancy of human nature, where human traits are considered distinct from animals and non-living entities (Haslam and Loughnan, 2014). In contrast, Eastern cultural contexts, particularly in China, often emphasize the inherent goodness of human nature. For example, the Chinese philosopher Mencius proposed that human nature is fundamentally good, which is expressed through four virtues: “compassion,” “sense of shame,” “courtesy,” and “sense of right and wrong” (Wang and Cui, 2008).

Despite these cultural differences, dehumanization, such as online bullying, has become an increasingly unavoidable issue in modern society. Therefore, there is a pressing need to conduct localized research on dehumanization in China.

The core of human nature lies in social connection (Haslam, 2021). Dehumanization, which involves the loss of human nature, leads to the animalization or objectification of individuals, weakening, alienating, and severing interpersonal connections. This phenomenon can originate as early as childhood (Kteily and Landry, 2022). Object relations theory, with attachment as a central concept, suggests that childhood development significantly affects adult achievements. Attachment refers to the interpersonal relationship patterns formed through an individual’s early interactions with their parents. These relatively stable interpersonal expectations, along with emotional and behavioral styles, play a key role throughout one’s life (Bowlby, 1982) and profoundly affect personal development and interpersonal relationships (Mikulincer and Shaver, 2012). Based on these premises, this study aimed to examine the association between attachment and dehumanization from the perspectives of both trait attachment and state attachment.

### 1.1 Adult attachment and attachment style

Based on Bowlby’s attachment theory, Hazan and Shaver (1987) proposed that adult romantic relationships are also attachment relationships, which is called adult attachment. Adults develop early internal working models that reflect their reactions to romantic relationships, influencing their behavior in attachment-related situations and demonstrating cross-temporal stability. From this perspective, attachment is often viewed as a stable personality trait, known as attachment style. Ainsworth et al. (2015) originally classified attachment styles into three categories: secure, anxious-resistant, and avoidant.

Subsequently, Bartholomew and Horowitz (1991) expanded on this by identifying two main dimensions within the attachment system: anxiety and avoidance. Anxiety reflects a fear of abandonment but with a strong desire for closeness, while avoidance indicates discomfort with intimacy and a strong desire for independence (Mikulincer and Shaver, 2012). High scores on these two dimensions are indicative of insecure attachment.

Furthermore, based on two dimensions and incorporating the self-other model, they have further proposed a four-category model of adult attachment styles, building upon the internal working models. In this model, a positive view of both self and others corresponds to low anxiety and low avoidance, termed as secure attachment. A positive self-view but a negative view of others corresponds to low anxiety and high avoidance, referred to as dismissive attachment. Conversely, a negative self-view but positive view of others aligns with high anxiety and low avoidance, known as preoccupied attachment. Finally, a negative view of both self and others corresponds to high anxiety and high avoidance, which is known as fearful attachment.

Brennan et al. (1998) developed the widely used Experiences of Close Relationships Scale (ECR), which measures attachment using the two dimensions of anxiety and avoidance, resulting in four orthogonal attachment types: high anxiety low avoidance, low anxiety low avoidance, high anxiety high avoidance, and low anxiety high avoidance.

For a long time, adult attachment styles have been controversial, particularly regarding whether attachment is best understood as dimensional or categorical. The dimensional approach tends to view attachment across different variables and dimensions, while the categorical approach focuses on individual variations.

The ECR scale adopts a variable-oriented method, which is useful for examining commonalities between variables but overlooks individual heterogeneity. In contrast, latent profile analysis (LPA) is a person-oriented approach that emphasizes individual differences by grouping research subjects into distinct categories and then exploring their shared characteristics.

LPA is also suitable for analyzing continuous observed variables and offers the advantage of reporting category probabilities (Wen et al., 2023). Therefore, this study will use a localized version of the ECR, utilizing LPA and an individual differences framework, to verify the structure of adult attachment styles based on two dimensions and four types.

### 1.2 Trait-based adult attachment and dehumanization

Dehumanization refers to the process of denying the humanity of others (Haslam et al., 2005). It is associated with behaviors, such as violence, racial discrimination, racial stereotyping, and the imitation of violent behaviors from games, which May reduce prosocial behaviors and increase antisocial behaviors. To investigate more general phenomena of individual conflict, Haslam et al. (2005) proposed a dual model of dehumanization. This model distinguishes between two forms: the dehumanization of unique human nature and the dehumanization of general human nature. When unique human nature is denied, individuals are perceived as animal-like (animalistic dehumanization). When general human nature is denied, individuals are seen as lacking cognitive flexibility and warmth (mechanistic dehumanization). Haslam also differentiated between self-dehumanization and other-dehumanization. Self-dehumanization refers to an individual’s recognition and evaluation of their own dehumanization, while other-dehumanization involves the evaluation of others as less human.

Dehumanization is rooted in interpersonal relationships (Maynard and Luft, 2023). Attachment, the emotional bond that connects individuals to others, not only benefits the individual but also promotes social interactions and prosocial behaviors. A lack of attachment can lead to social alienation and a denial of one’s ability to form social connections.

Unhealthy social relationships, such as those marked by frustration, neglect, or a lack of belonging, can result in dehumanization (Jenkins et al., 2023; Zhang and Chen, 2024). Insecure attachment patterns, often resulting from long-term neglect or harm, and low self-esteem in social relationships can exacerbate these issues. Although the relationship between dehumanization and adult attachment is not yet fully understood, early and lifelong attachment patterns May be closely linked to dehumanization.

Therefore, this study aims to further investigate whether different attachment types significantly influence dehumanization, especially whether insecure attachments lead to an increase in dehumanization. Understanding this connection is essential for identifying the factors that trigger dehumanization and could provide new strategies for its prevention and intervention.

### 1.3 State adult attachment and dehumanization

Attachment styles have traditionally been conceptualized as stable personality traits (Gillath et al., 2009). However, they can also evolve and adapt as new relationships and experiences emerge, with fluctuations occurring independently of trait-based attachment styles (Davila and Sargent, 2003). Relationships in specific contexts can activate specific attachment schemas. Specific contexts can activate particular attachment schemas, temporarily overriding an individual’s trait-based attachment style and influencing their perceptions, expectations, and behaviors (Mikulincer and Shaver, 2005). This context-specific attachment response is referred to as state attachment. In view of the fluidity of attachment types, attachment priming is a method used to activate the attachment system under certain conditions and examine an individual’s current attachment status and its influencing factors, such as state attachment.

A commonly used method for this is Secure Attachment Priming (SAP), which involves presenting secure attachment-related stimuli or asking participants to imagine or recall secure attachment-related feelings so that individuals can temporarily experience a series of positive responses associated with the secure attachment, such as reduced threats, relief from distress, a sense of love, comfort, and attachment security (Mikulincer et al., 2001). In contrast, insecure attachment leads to poor social relationships, reduced empathy, negative evaluations of others, and reduced prosocial behavior (e.g., DeWall and Baumeister, 2006; Twenge et al., 2007).

Therefore, it can be inferred that attachment priming, by enhancing an individual’s state of secure attachment, can increase their willingness to maintain stable relationships, show greater attention to others’ needs, and promote prosocial behaviors (Zhang et al., 2015; Jia et al., 2020; Scatolon et al., 2023), thereby reducing the level of dehumanization. If this inference is proven, it provides a new perspective and practical approach for preventing and intervening in dehumanization—suggesting that interventions aimed at improving individuals’ attachment levels could effectively reduce dehumanization.

### 1.4 The present study

In summary, this study aims to explore the relationship between adult attachment and dehumanization from the perspectives of both trait adult attachment and state adult attachment. Study 1 uses latent profile analysis to verify the four types of adult attachment and examines the differences in how each type affects dehumanization. Study 2 aims to explore the relationship between state adult attachment and dehumanization by priming participants’ sense of security through a recall writing task. To further clarify the analysis, this study proposes the following research hypotheses:

*H1:* There are four latent categories of adult attachment among Chinese college students.

*H2:* Different latent categories of adult attachment have varying effects on dehumanization.

*H3:* State adult attachment significantly predicts dehumanization.

## 2 Study 1

### 2.1 Method

#### 2.1.1 Participants

A total of 979 questionnaires were distributed to university students from various regions in China via the online platform Questionnaire Star. After excluding invalid responses—those with standardized scores beyond ±3 standard deviations, excessively long or short completion times, or indications of careless answers—the final sample consisted of 705 valid questionnaires. Among the respondents, 260 respondents were men (36.9%) and 445 respondents were women (63.1%). The academic year distribution was as follows: 131 freshmen (18.6%), 210 sophomores (29.8%), 152 juniors (21.6%), 106 seniors (15.0%), and 106 graduate students (15.0%).

Additionally, 297 respondents (42.1%) were only children, and 408 respondents (57.9) had siblings. Of these respondents, 367 of them (52.1%) came from urban families, while 338 of the respondents (47.9%) were from rural families (47.9%). The study was approved by the academic committee of Anhui Normal University, ensuring that participants were informed about the research purpose, voluntary participation, and anonymity. Each participant received compensation upon completing the questionnaire.

#### 2.1.2 Measures

##### 2.1.2.1 Adult attachment

Adult attachment was measured using the Chinese version of the Experiences of Close Relationships (ECR) Scale, originally developed by Brennan et al. (1998) and revised by Li and Kato (2006). This scale consists of 36 items rated on a 7-point Likert scale (1 = strongly disagree, 7 = strongly agree) and assesses two dimensions: anxiety and avoidance. Each dimension has three sub-dimensions, with higher scores indicating greater levels of the corresponding attachment type. An example item for the avoidance dimension is, “When my partner starts to get close to me, I find myself pulling away.”

An example item for the anxiety dimension is, “I am very concerned about my romantic relationships.” The Chinese version of the ECR demonstrates good internal consistency, with Cronbach’s alpha coefficients of 0.82 and 0.77 for the avoidance and anxiety subscales, respectively. In this study, the Cronbach’s alpha coefficients were 0.85 for avoidance, 0.82 for anxiety, and 0.91 for the total scale.

##### 2.1.2.2 Dehumanization

We used the Chinese version of the Perceptions of Humanness Scale to measure dehumanization, which was originally developed by Bastian et al. (2012) and revised by Chen et al. (2014). The scale consists of 16 items rated on a 7-point Likert scale (1 = strongly disagree, 7 = strongly agree). It includes two subscales, self-humanization, and other-humanization, each with two sub-dimensions: human nature and human uniqueness.

An example item for the human nature sub-dimension is, “I feel that my thinking is broad, and I can consider some things more clearly.” An example item for the human uniqueness sub-dimension is, “I feel that I lack self-control and behave like an animal.” Research has shown that the dehumanization tendencies measured by this scale are stable over time (Lantos, 2023). This scale has been widely used in dehumanization research in China (e.g., Chen et al., 2014; Li et al., 2018). In this study, Cronbach’s alpha coefficients were 0.80 for self-humanization, 0.82 for other-humanization, and 0.87 for the total scale.

### 2.2 Statistical methods

According to Vermunt (2010) and Wen et al. (2023), the principles and procedures of latent profile analysis should be conducted using Mplus, with subsequent analysis conducted using SPSS analysis. Following this guidance, we first calculated descriptive statistics and Pearson correlation coefficients for the study variables using SPSS 28.0. Then, a latent profile analysis of adult attachment was conducted using Mplus 8.3 to explore the latent categories and distribution. Subsequently, we conducted a series of analyses of variance (ANOVA) using SPSS 28.0 to examine the effects of different levels of adult attachment on dehumanization. Finally, binary logistic regression analysis was conducted to investigate the predictive effects of gender, grade, only-child status, and family origin on adult attachment among college students.

### 2.3 Results

#### 2.3.1 Common method bias

Using Harman’s single-factor test, the results showed that eight factors had eigenvalues greater than 1, and the variance explained by the first factor was 22.91%, which is below the 40% threshold, indicating no severe common method bias.

#### 2.3.2 Descriptive statistics and correlation analysis

With gender, grade, only-child status, and family origin variables as controlled variables, avoidance, anxiety, self-dehumanization, and other-dehumanization were found to be significantly positively correlated in pairs (Table 1).

**Table 1:** Descriptive statistics and correlations of the main variables (*N* = 705).

Variable	*M ± SD*	1	2	3	4
1. Avoidance	10.11 ± 2.98				
2. Anxiety	11.77 ± 3.19	0.21^***^			
3. Self-dehumanization	24.33 ± 7.43	0.43^***^	0.38^***^		
4. Other-dehumanization	25.29 ± 7.32	0.36^***^	0.30^***^	0.55^***^	
5. Dehumanization	49.62 ± 12.97	0.45^***^	0.39^***^	0.88^***^	0.88^***^

^***^p < 0.001.

#### 2.3.3 Latent profile analysis

We used the six sub-dimensions of the attachment scale as indicators, with dimensions 1, 2, and 3 representing avoidance and dimensions 4, 5, and 6 representing anxiety. Latent profile analysis was conducted to determine the number of attachment categories, ranging from 1 to 6. For the best-class solution, we considered the following model fit indices: (1) lower Akaike information criterion (AIC), Bayesian information criterion (BIC), and sample-size-adjusted BIC (SSA-BIC), (2) an entropy value of ≥0.8, and (3) a significant Lo–Mendell-Ruben (LMR) and bootstrap likelihood ratio test (BLRT) (Lubke and Muthén, 2007).

The results showed (Table 2) that as the number of categories increased from 1 to 6, AIC, BIC, and SSA-BIC values gradually decreased. Additionally, BLRT and LMR were significant for the four-category model, with an entropy value of 0.86 (> 0.8), indicating a better classification effect. Therefore, adult attachment could be classified into four categories.

**Table 2:** Model fit indices for each LPA solution.

Model	AIC	BIC	SSA-BIC	LMR(p)	BLRT(p)	Entropy	Category probability
1–group	4589.23	4607.46	4594.76	–––	–––	–––	–––
2–groups	4222.71	4254.62	4232.39	<0.001	<0.001	0.74	0.50/0.50
3–groups	11879.62	11998.13	11915.58	0.0024	<0.001	0.84	0.51/0.26/0.23
**4–groups**	**11521.08**	**11671.50**	**11566.72**	**0.0031**	**<0.001**	**0.86**	**0.22/0.26/0.45/0.07**
5–groups	11272.70	11455.03	11328.02	0.029	<0.001	0.84	0.20/0.23/0.31/0.06/0.19
6–groups	11077.21	11291.44	11142.21	0.0035	<0.001	0.85	0.18/0.06/0.08/0.19/0.29/0.20

AIC, Akaike’s information criterion; BIC, Bayesian information criterion; SSA-BIC, sample-adjusted BIC; LMR, Lo–Mendell-Ruben; BLRT, bootstrap likelihood ratio test. In bold, we report the best LPA solution.

**Figure 1:**
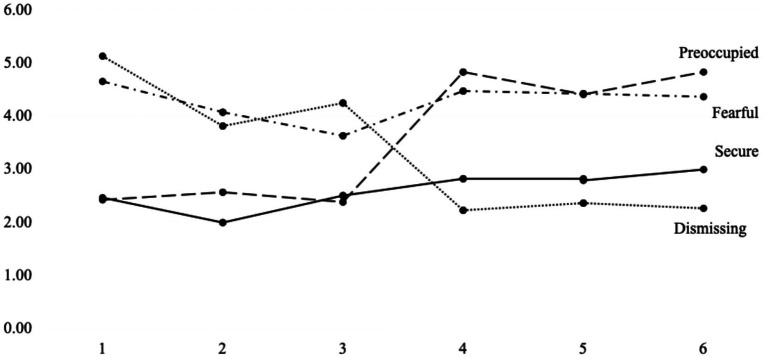
The results of latent profile analysis (LPA) with four-class solution.

The latent profile plot (Figure 1) reveals that the four categories correspond to the four types of attachment proposed by Bartholomew and Horowitz: preoccupied (low avoidance-high anxiety), secure (low avoidance-low anxiety), fearful (high avoidance-high anxiety), and dismissing (high avoidance-low anxiety) (Bartholomew and Horowitz, 1991). The proportions of these four types were 0.22, 0.26, 0.45, and 0.07, respectively, with insecure attachments (including fearful, preoccupied, and dismissing) accounting for 78% of the total.

#### 2.3.4 ANOVA

We conducted a one-way ANOVA to examine differences in self-dehumanization and other-dehumanization across the latent classes of adult attachment. The results showed that the latent classes of adult attachment had a significant effect on self-dehumanization (*F* (3, 705) = 64.78, *p <* 0.001). The *post-hoc* analysis found that the fearful group had the highest self-dehumanization score and was significantly different from both the secure and preoccupied groups (*p* < 0.001). The secure, fearful, and dismissive groups differed significantly in self-dehumanization (*p <* 0.001). The fearful and dismissive differed significantly in self-dehumanization (*p* < 0.05). However, the preoccupied and dismissive groups did not differ significantly in self-dehumanization (*p* > 0.05). Furthermore, the four attachment classes also showed significant differences in other-dehumanization [*F* (3, 705) = 42.16, *p <* 0.001].

Similar to the self-dehumanization findings, the *post-hoc* analysis revealed that the fearful group had the highest score for other-dehumanization and differed significantly from the secure, preoccupied, and dismissive groups (*p <* 0.001 for all). Additionally, the preoccupied and fearful groups differed significantly in other-dehumanization (*p* < 0.05). However, no significant difference was found between the preoccupied and dismissive groups in other-dehumanization (*p* > 0.05) (Table 3).

**Table 3:** Differences in self-dehumanization and other-dehumanization scores for adult attachment categories.

Implicit variable	Preoccupied (C1)	Secure (C2)	Fearful (C3)	Dismissing (C4)	*F*	*Post hoc* comparison
Self-dehumanization	24.18 ± 6.83	18.95 ± 6.87	27.44 ± 6.09	24.46 ± 7.88	64.78***	C1 > C2; V3 > C1. C4 > C1; C3 > C4
Other-dehumanization	25.80 ± 6.65	20.61 ± 7.65	27.58 ± 5.96	26.09 ± 8.33	42.16^***^	C1 > C2; C3 > C1. C4 > C1; C3 > C4

^***^p < 0.001.

#### 2.3.5 Logistic regression analysis

To further explore the relationship between sub-types of adult attachment and both self-dehumanization and other-dehumanization, self-dehumanization was divided into two groups based on the average score: Group 1 (high self-dehumanization, > 24) and Group 2 (low self-dehumanization, ≤ 24). Similarly, other-dehumanization was divided into two groups: Group 3 (high other-dehumanization, > 26) and Group 4 (low other-dehumanization, ≤ 26), which were also based on the average score.

Then, we used binary logistic regression to assess the impact of different categories of adult attachment on self-dehumanization and other-dehumanization, controlling for variables such as gender, grade, only-child status, and family origin.

The results for the self-dehumanization model indicated that family origin (*p* < 0.05) and the latent categories of adult attachment (*p <* 0.001) were significant. Urban university students had a lower probability (0.65 times) of self-dehumanization compared to rural university students (*p* < 0.05). The secure group had a 0.30 times lower probability of self-dehumanization compared to the dismissive group (*p <* 0.001), while the fearful had a 2.34 times higher probability of self-dehumanization compared to the dismissive group (*p <* 0.001).

For the other-dehumanization model, gender (*p* < 0.05), grade (*p* < 0.01), and the latent categories of adult attachment (*p <* 0.001) were significant. Men had a 1.58 times higher probability of other-dehumanization compared to women (*p* < 0.05). Junior students had a 0.52 times lower probability of other-dehumanization compared to graduate students (*p* < 0.05). The secure group had a 0.33 times lower probability of other-dehumanization compared to the dismissive group (*p* < 0.01), while the fearful group had a 2.06 times higher probability of other-dehumanization compared to the dismissive group (*p* < 0.05) (Table 4).

**Table 4:** Logistic regression analysis.

Variant	Self-dehumanization (high vs. low)			Other-dehumanization (high vs. low)		
	*B*	Wald	OR (95% CI)	*B*	Wald	OR (95% CI)
**Sexes**						
Male	0.08	0.22	1.09 [0.77, 1.54]	0.46^*^	6.70	1.58 [1.12, 2.24]
Female	0			0		
**Grade**						
Freshman	−0.23	0.64	0.79 [0.45, 1.40]	0.27	0.87	1.31 [0.75, 2.28]
Sophomore	−0.35	1.78	0.71 [0.42, 1.18]	−0.17	0.42	0.85 [0.51, 1.40]
Junior	−0.50	3.17	0.61 [0.35, 1.05]	−0.66^*^	5.72	0.52 [0.30, 0.89]
Senior	−0.51	2.84	0.60 [0.33, 1.09]	0.05	0.03	1.05 [0.59, 1.88]
Postgraduate	0			0		
**Only child**						
Yes	−0.28	2.39	0.76 [0.53, 1.08]	0.11	0.34	1.11 [0.78, 1.58]
No	0			0		
**Family origin**						
Urban areas	−0.44^*^	6.01	0.65 [0.46, 0.92]	−0.15	0.67	0.87 [0.61, 1.22]
Rural areas	0			0		
**Adult attachment latent category**						
Preoccupied	−0.02	0.002	0.99 [0.50, 1.95]	0.06	0.03	1.06 [0.54, 2.10]
Secure	−1.22^***^	11.55	0.30 [0.15, 0.60]	−1.12^**^	9.97	0.33 [0.16, 0.65]
Fearful	0.85^***^	6.64	2.34 [1.23, 4.47]	0.72^*^	4.82	2.06 [1.08, 3.92]
Dismissive	0			0		

^*^p < 0.05; ^**^p < 0.01; ^***^p < 0.001.

## 3 Study 2

### 3.1 Methods

#### 3.1.1 Participants and procedures

A total of 330 Chinese students from various universities were recruited for the online experiment. This study was approved by the Academic Committee of Anhui Normal University. Before the experiment began, researchers explained the study’s purpose, the voluntary nature of participation, and safety considerations to the participants. After excluding invalid and missing data, the final effective sample size was 281. Among the participants, 148 were men (52.7%) and 133 were women (47.3%). The distribution of student numbers across academic years was as follows: 42 freshmen (14.9%), 99 sophomores (35.2%), 99 juniors (35.2%), 31 seniors (11.0%), and 10 postgraduates (3.6%). A total of 143 participants (50.9%) were only children, while 138 (49.1%) had siblings. Additionally, 114 participants (40.6%) came from rural areas, and 167 (59.4%) came from urban areas. All participants had normal vision and were right-handed.

Participants were randomly divided into two groups: Group 1 (secure priming) had 143 participants, and Group 2 (neutral priming) had 138 participants. Different instructions were provided to the two groups based on the materials for the recall writing task. After 2 min, the participants were asked to complete five items related to the recall writing task to assess the effectiveness of secure attachment priming and writing. Finally, the participants completed the State Adult Attachment Scale and the Humanization Perception Scale. Each participant received compensation after the experiment.

#### 3.1.2 Measures

##### 3.1.2.1 Recall writing task priming materials

Referring to the study by Li et al. (2016), participants were primed with either secure attachment or neutral attachment by recalling a writing task. Instructions for Group 1 (secure priming) were: “Please recall a person intimate with you…” followed by “Carefully recall their appearance and the feelings when you interact with them, then complete the following items.” For Group 2 (neutral priming), the instructions were: “Please recall an unfamiliar person…” followed by “Carefully recall their appearance and the feelings when you interact with them, then complete the following items.” After receiving these prompts, the participants were given 2 min to recall and then complete the following four items: (1) What is the appearance of the person you imagine? What facial features does he/she have? (2) What is the relationship between the person you imagine and yourself? How did you come to know he/she? (3) What is the general content of the event you are recalling? (4) How would you feel if this person were with you right now?

The participants were instructed to recall as carefully as possible and provide detailed responses to these questions. After completing the five items, the participants were informed to continue imagining the person they recalled to complete the subsequent attachment security validity and writing validity tasks. These five items were designed solely to facilitate recall and were not included in the data analysis.

##### 3.1.2.2 Attachment security activation validity assessment tool

The validity of attachment security activation was assessed using the tool developed by Li et al. (2016), with a total score of ≥4 indicating effectiveness. This tool evaluates feelings of secure attachment with five descriptive words: secure, warm, caring, supportive, and intimate. After completing the recall writing task, the participants rated their feelings on a 5-point scale (1 = not at all, 5 = very much) for each word. An example item is as follows: “To what extent do I feel warmth when I imagine him/her being with me?” In Li et al.’s study, Cronbach’s alpha coefficient for these five items was 0.96, while in the present study, it was 0.89.

##### 3.1.2.3 Writing effectiveness assessment tool

The validity of the writing task was assessed using a tool developed by Mikulincer et al. (2001) and adapted into Chinese by Li et al. (2016). Effective writing encompasses both vividness and ease of comprehension, each rated on a 5-point scale (1 = very vivid/easy, 5 = not vivid/easy at all). The items are as follows: “The vividness of the writing content during the recall writing process is” and “The ease of writing during the recall writing process is.”

##### 3.1.2.4 State adult attachment measure (SAAM)

The State Adult Attachment Measure (SAAM), developed by Gillath et al. (2009) and revised into Chinese by Ma et al. (2012), is a 21-item scale that measures three dimensions of attachment: security, avoidance, and anxiety. Higher scores on these dimensions reflect the corresponding attachment states. For example, a higher score on the security dimension indicates a secure attachment state, while higher scores on the avoidance and anxiety dimensions reflect greater levels of avoidance and anxiety in attachment, respectively. Examples include “I feel that others care about me” (for security) and “I am afraid that someone will get too close to me” (for anxiety). Both structural validity and test–retest reliability assessments confirm that the Chinese version of SAAM has strong psychometric properties. In this study, Cronbach’s alpha coefficients for the three dimensions were 0.86 (security), 0.91 (avoidance), and 0.85 (anxiety), respectively, with an overall value of 0.83.

##### 3.1.2.5 Humanization perception scale

As in Study 1, the Cronbach’s alpha coefficients for the two subscales were 0.87 and 0.87, respectively, with a total scale reliability of 0.92.

### 3.2 Statistical methods

Descriptive statistics and correlation analysis were conducted for each variable using SPSS 28.0. Independent sample *t*-tests were performed to compare differences in attachment security and recall writing task effectiveness scores between the two groups. Then, attachment types were classified into three types of categories, and a multifactor ANOVA was conducted to examine the differences in dehumanization across the three attachment types between the two groups, followed by a simple effects test. Finally, we used regression analysis to assess the predictive effect of state adult attachment on dehumanization.

### 3.3 Results

#### 3.3.1 Common method bias

Harman’s single factor test was used to assess common method bias. The results showed that eight factors had eigenvalues greater than 1, and the variance explained by the first factor was 27.43%, which is below the standard threshold of 40%, indicating that there was no excessive common method bias in this study.

#### 3.3.2 Descriptive statistics and correlation analysis

Descriptive statistics and correlation analysis for each variable are presented in Table 5. The correlations between security and avoidance, anxiety and self-dehumanization, and anxiety and other-dehumanization were not significant. However, significant correlations were observed between other pairs.

**Table 5:** Descriptive statistics and correlations of the main variables (*N* = 281).

	*M*	*SD*	1	2	3	4
1. Security	5.58	0.73				
2. Avoidance	3.87	1.36	−0.12			
3. Anxiety	5.40	0.92	0.47^***^	0.18^*^		
4. Self-dehumanization	3.19	0.99	−0.39^***^	0.56^***^	−0.11	
5. Other-dehumanization	3.07	0.96	−0.35^***^	0.55^***^	−0.12	0.70^***^

^*^p < 0.05, ^***^p < 0.001.

#### 3.3.3 Priming effectiveness test

The security scores for Group 1 (secure priming) were significantly higher than those for Group 2 (neutral priming) (*t* = 5.81, *p* < 0.001), with an average score greater than 4. However, there were no significant differences between the two groups in terms of vividness (*p* > 0.05) and ease (*p* > 0.05) (Table 6), indicating that the priming was effective.

**Table 6:** Comparison of the two groups’ scores on the initiation validity test index (M ± SD).

Variant	Secure priming group (*n* = 143)	Neutral priming group (*n* = 138)	*t*	*p*
Security	4.39 ± 0.49	3.92 ± 0.85	5.81	0.000
Writing vividness	3.73 ± 0.75	3.83 ± 0.80	−1.14	0.255
Ease of writing	3.69 ± 0.88	3.88 ± 0.96	−1.68	0.093

#### 3.3.4 Difference test

Based on previous research on attachment type classification (Chavis and Kisley, 2012; Dan and Raz, 2012; Zilber et al., 2007), participants with scores higher than the average for security and lower than the averages for avoidance and anxiety were classified as secure. Those with scores lower than the averages for security and anxiety but higher than the average for avoidance were classified as avoidants. Participants with lower scores than the average for security and avoidance but higher than the average for anxiety were classified as anxious. Self-dehumanization and other-dehumanization scores were compared among the attachment types in both groups (Table 7).

**Table 7:** Comparison of scores on self-dehumanization and other-dehumanization between the two groups (M ± SD).

	Secure priming group (*n* = 57)			Neutral priming group (*n* = 46)		
	Security (*n* = 26)	Avoidance (*n* = 13)	Anxiety (*n* = 18)	Security (*n* = 17)	Avoidance (*n* = 14)	Anxiety (*n* = 15)
Self-dehumanization	2.48 ± 0.95	3.90 ± 0.49	1.95 ± 0.74	2.98 ± 0.95	4.06 ± 0.65	3.02 ± 0.89
Other-dehumanization	2.98 ± 0.95	4.10 ± 0.42	1.83 ± 0.65	2.93 ± 0.95	3.93 ± 0.57	2.69 ± 0.47

A multifactorial analysis of variance was conducted, with the variables being group (secure activation vs. neutral activation) and attachment type (secure, avoidant, anxious). The dependent variables were self-dehumanization and other-dehumanization. The results (see Table 8) indicated that both group [*F* (1, 97) = 12.011, *p* < 0.01] and attachment type [*F* (2, 97) = 28.022, *p* < 0.001] had significant effects on self-dehumanization, but the interaction between group and attachment type on self-dehumanization was not significant [*F* (2, 97) = 2.401, *p* > 0.05]. For other-dehumanization, both groups [*F* (1, 97) = 7.461, *p* < 0.01] and attachment type [*F* (2, 97) = 49.731, *p* < 0.001] showed significant effects, and their interaction was also significant [*F* (2, 97) = 4.027, *p* < 0.05].

**Table 8:** Tests of differences between groups and attachment types in self-dehumanization and other-dehumanization.

	Self-dehumanization			Other-dehumanization		
	*F*	*p*	*η* ^2^	*F*	*p*	*η* ^2^
Groups	12.011	0.001	0.110	7.461	0.007	0.071
Attachment styles	28.022	0.000	0.366	49.731	0.000	0.506
Groups * attachment styles	2.401	0.096	0.047	4.027	0.021	0.077

Furthermore, simple effects tests revealed a significant difference in self-dehumanization among participants in the attachment security priming group across different attachment types [*F* (2, 97) = 22.291, *p* < 0.001, *η*^2^ = 0.315]. The avoidance group scored significantly higher in self-dehumanization compared to the security and anxiety groups (*p* < 0.001), while no significant difference was found between the anxiety and security groups (*p* > 0.05). Similarly, a significant difference in self-dehumanization was found among participants in the neutral priming group across different attachment types [*F* (2, 97) = 8.202, *p* < 0.01, *η*^2^ = 0.145]. The avoidance group had a higher self-dehumanization score than the security and anxiety groups (*p* < 0.001), but no significant difference was observed between the anxiety and security groups (*p* > 0.05).

Attachment Security Initiation Group showed significant differences in other-dehumanization among different attachment types [*F* (2, 97) = 41.205, *p* < 0.001, *η*^2^ = 0.459]. The avoidance group scored significantly higher in other-dehumanization compared to the security and anxiety groups (*p* < 0.001), and the security group scored higher than the anxiety group (*p* < 0.05). Similarly, the neutral priming group also showed significant differences in other-dehumanization across different attachment types [*F* (2, 97) = 12.828, *p* < 0.001, *η*^2^ = 0.209]. The avoidance group scored significantly higher than both the security and anxiety groupsin other-dehumanization (*p* < 0.001), with no significant difference between the security and anxiety groups (*p* > 0.05).

#### 3.3.5 Regression analysis

A linear regression analysis was conducted with attachment styles (secure, avoidant, and anxious) as independent variables and self-dehumanization and other-dehumanization as dependent variables. Both security and avoidance significantly predicted self-dehumanization (security: *t* = −5.943, *p* < 0.001; avoidance: *t* = 11.135, *p* < 0.001). Similarly, both security and avoidance also significantly predicted other-dehumanization (security: *t* = −4.543, *p* < 0.001; avoidance: *t* = 11.324, *p* < 0.001). However, anxiety did not have a significant impact on either self-dehumanization or other-dehumanization (see Table 9).

**Table 9:** Linear regression of attachment type with self-dehumanization and other-dehumanization.

	Self-dehumanization				Other-dehumanization			
	*B*	SE	Beta	*t*	*B*	SE	Beta	*t*
Constant	4.424	0.397		11.141^***^	3.954	0.393		10.066^***^
Security	−0.423	0.071	−0.312	−5.943^***^	−0.320	0.070	−0.244	−4.543^***^
Avoidance	0.384	0.034	0.528	11.135^***^	0.386	0.034	0.548	11.324^***^
Anxiety	−0.066	0.056	−0.061	−1.162	−0.109	0.056	−0.104	−1.948

^***^p < 0.001.

## 4 General discussion

### 4.1 Potential categories of adult attachment

The results of the latent profile analysis confirmed Hypothesis 1, indicating that categorizing the participants into four types is appropriate and supports the four-type attachment theory (Bartholomew and Horowitz, 1991). These four types are preoccupied (low avoidance-high anxiety), secure (low avoidance-low anxiety), fearful (high avoidance-high anxiety), and dismissive (high avoidance-low anxiety). The analysis also revealed a high proportion of insecure attachments, with the fearful type being the most prevalent among insecure attachment types. This finding aligns with existing research in China (e.g., Xu et al., 2016; Wei and Hou, 2009).

However, Western research presents different distribution characteristics. A meta-analysis conducted in the US found that the proportion of secure attachment types among American college students is the highest (Konrath et al., 2014). This disparity May be attributed to cultural differences. In China, the Confucian moral system and collectivist beliefs emphasize restraint, subtlety, and caution, discouraging direct emotional expression and intense physical contact. Consequently, Chinese students, despite emotionally desiring close relationships, May experience considerable hesitancy and distrust in their interactions, leading to lower levels of attachment (Xu and Pang, 2014; Li, 2024).

Overall, compared to American students, the attachment patterns of Chinese students are concerning, with insecure attachment negatively affecting various psychological functions. The fact that over half of college students exhibit insecure attachment is a matter of concern. These findings provide theoretical support for the four-type adult attachment model in China and highlight the need for further research and intervention efforts.

### 4.2 Relationship between potential categories of adult attachment and dehumanization

The results of this study reveal that secure attachment is associated with lower levels of dehumanization compared to insecure attachment, with the fearful attachment style showing the highest levels of dehumanization. These results confirm Hypothesis 2. Specifically, secure attachment is characterized by positive psychological models, confidence in oneself and others, and healthy self-esteem, without extreme interpersonal barriers. Previous research has also indicated that secure attachment May be a key factor in reducing dehumanization (Kteily and Landry, 2022).

In contrast, fearful attachment (high avoidance and high anxiety) represents a severe form of insecure attachment. Individuals with fearful attachment hold negative models of both themselves and others, marked by feelings of worthlessness, distrust, and rejection (Bartholomew and Horowitz, 1991). This attachment style leads to negative evaluations of both self and others and fosters negative attitudes toward humanity (Karantzas et al., 2022; Karantzas et al., 2023).

Studies on dehumanization in interpersonal and attachment relationships (Bastian et al., 2012; Pizzirani et al., 2021) found that dehumanization is usually associated with avoidant emotions (e.g., numbness, sadness) and behaviors indicative of poor relationship quality (Kteily and Landry, 2022; Ariño-Mateo et al., 2022). Therefore, insecure attachment is more likely to be associated with high levels of dehumanization.

Logistic regression analysis of demographic factors revealed that urban university students have a lower probability of self-dehumanization compared to their rural counterparts. This difference May be attributed to the fact that urban students generally experience more social interactions and receive more positive feedback in their developmental environment, which fosters higher self-identity and self-esteem (Mao et al., 2021). As a result, urban students tend to exhibit lower levels of self-dehumanization.

Additionally, the probability of other-dehumanization was found to be higher among male students compared to female students. This aligns with traditional Chinese cultural expectations, where men are often expected to be responsible, strong, proactive, and competitive. These societal pressures May lead men to exhibit higher levels of aggression and competitiveness, contributing to more negative perceptions of others and, consequently, higher levels of dehumanization.

The results also indicate that third-year students have a lower probability of other-dehumanization compared to graduate students. One possible explanation is that graduate students, having reached higher educational levels, May develop a stronger sense of superiority, leading to more negative evaluations of others, such as perceiving them as less mature or intelligent, which could lead to higher levels of other-dehumanization. In contrast, third-year students, who are more mature than first- and second-year students, May have a more developed and positive attitude toward others. However, this aspect warrants further detailed investigation.

### 4.3 Relationship between state adult attachment and dehumanization

These findings from the ANOVA and regression analysis partially corroborate previous studies. For example, Mikulincer and Shaver (2005) found that when primed with the names of secure attachment figures, participants reported more helping behaviors. The reason could be feelings of support and care in social relationships, which can evoke a sense of interpersonal security. Such feelings reduce the perceived differences between in-groups and out-groups, fostering a sense of shared humanity and lowering dehumanization.

Secure attachment priming can encourage more positive, open, and flexible information processing, making individuals more active in interpersonal interactions and more willing to maintain stable relationships with others. It inclines them to adopt positive interpersonal strategies, which reduces egocentrism, increases prosocial behavior, and decreases aggression, prejudice, and discrimination (Jia et al., 2020).

Therefore, secure attachment priming significantly decreased dehumanization scores.

The interaction between group and attachment type had a significant impact on other-dehumanization. This effect May be due to the secure activation process, which prompts individuals to recall positive and warm experiences related to others, fostering a more positive view and attitude toward them, thereby reducing other-dehumanization. However, the effect of the interaction on self-dehumanization was not significant, suggesting that the activation process did not significantly alter individuals’ self-perceptions, as the focus was more on the feelings elicited by others rather than on self-evaluation, resulting in no notable change in self-dehumanization levels.

In contrast, individuals with avoidant attachment typically exhibit indifference and rejection. They often cope with interpersonal problems through blame, emotional coldness, and withdrawal, devaluing or minimizing the importance of relationships (Mikulincer and Shaver, 2005). Such behavior can lead to prejudice, denial of others, and consequently, higher levels of dehumanization. Notably, anxiety under secure priming resulted in lower levels of other-dehumanization compared to secure attachment. A possible explanation May be that individuals with anxiety tend to hold a positive view of others, which is enhanced under secure priming, leading to lower levels of other-dehumanization.

However, the regression analysis revealed that anxiety did not significantly predict dehumanization. A possible reason may be the inherent contradictions and inconsistencies associated with anxiety. On the one hand, individuals with anxiety have a strong desire for interpersonal connection, but on the other hand, they tend to exaggerate potential negative outcomes, potentially leading to negative emotional responses, such as anger, hurt, and excessive rumination (Peng et al., 2023). These conflicting tendencies May counterbalance each other, reducing the overall impact of anxiety traits on dehumanization. As a result, anxiety traits May not significantly predict dehumanization.

## 5 Limitations and further directions

Although this study is the first to explore the relationship between adult attachment and dehumanization and to validate the predictive role of adult attachment in dehumanization, several limitations should be considered. First, the reliance on self-report questionnaires could have introduced social desirability bias, potentially leading to deviations from actual conditions. Future research might benefit from employing experimental methods or other approaches to address this issue.

Second, understanding the pathways through which adult attachment influences dehumanization is crucial for developing prevention and intervention strategies. Further research is required to uncover the underlying mechanisms that link adult attachment to dehumanization. In addition to attachment, other predictive variables for dehumanization, such as social class, ethnicity, and related factors, should also be considered as directions for future research.

Additionally, exploring the longitudinal relationship between attachment and dehumanization, considering the developmental changes from childhood to adulthood, could provide more comprehensive and systematic conclusions.

Finally, since this study was conducted in China, cultural differences between Eastern and Western perspectives—particularly in terms of dehumanization—May lead to different findings in other countries or regions. Further research in diverse cultural contexts is warranted to gain a broader understanding of these dynamics.

## 6 Conclusion

This study investigates the relationship between adult attachment and dehumanization from both trait and state attachment perspectives. First, a latent profile analysis was conducted to validate four types of trait-based adult attachment—preoccupied, secure, fearful, and dismissing—and to examine the significant differences in how these attachment types influence dehumanization. Additionally, it also examined the impact of demographic variables on these latent categories. Subsequently, a secure attachment priming experiment was conducted to assess the impact of three types of state-based attachments—security, avoidance, and anxiety—on dehumanization. The findings revealed that both security and avoidance types significantly predict dehumanization. By linking attachment theory with dehumanization, this study not only confirms the relationship between adult attachment and dehumanization but also provides a novel perspective and approach for the prevention and intervention of dehumanization. Furthermore, it contributes to the localization of dehumanization research in China.

## Data Availability

The raw data supporting the conclusions of this article will be made available by the authors, without undue reservation.
